# Nanoscale rheology at solid-complex fluid interfaces

**DOI:** 10.1038/s41598-017-04294-4

**Published:** 2017-06-30

**Authors:** Sebastian Jaksch, Olaf Holderer, Manuchar Gvaramia, Michael Ohl, Michael Monkenbusch, Henrich Frielinghaus

**Affiliations:** 1 Forschungszentrum Jülich GmbH, JCNS at Heinz Maier-Leibnitz Zentrum, Lichtenberstraße 1, 85747 Garching, Germany; 2 Forschungszentrum Jülich GmbH, JCNS at SNS-Oak Ridge National Laboratory (ORNL), 1 Bethel Valley Road, Oak Ridge, TN 37831 USA; 3 Forschungszentrum Jülich GmbH, Jülich Centre for Neutron Science JCNS, Wilhelm-Johnen-Straße, 52428 Jülich, Germany

## Abstract

Here we present an approach to measure dynamic membrane properties of phospholipid membranes close to an interface. As an example we show results of the membrane dynamics of a phospholipid membrane multilayer-stack on a solid substrate (silicon). On this sample we were able to measure local interaction and friction parameters using *Grazing Incidence Neutron Spin Echo Spectroscopy* (GINSES), where an evanescent neutron wave probes the fluctuations close to a rigid interface. With this method it is possible to access length scales in the nano to micrometer region as well as energies in the *μeV* range. Using a new neutron resonator structure we achieved the required intensity gain for this experiment. During our investigations we found an excitation mode of the phospholipid membrane that has not been reported previously and only became visible using the new methodology. We speculate that the energy transported by that undulation can also serve to distribute energy over a larger area of the membrane, stabilizing it. This new methodology has the capability to probe the viscoelastic effects of biological membranes, becoming a new tool for tribology on the nanoscale and has allowed the observation of the hitherto invisible property of phospholipid membranes using neutrons.

## Introduction

A detailed understanding of the rheology and friction at interfaces is of vital importance for a wide range of biological and medical applications, such as lubrication and coating in mammalian joints^1^, diffusion properties of membranes for drug delivery^2^ or general permeability considerations for cellular membranes^3^. The links between the cellular behaviour and properties of phospholipid membranes are treated in a review by Tanaka^4^. Investigations of such systems have been carried out using a wide range of methods, such as light-scattering^3^, atomic force microscopy (AFM)^5^, X-ray scattering^6–12^ as well as elastic^10, 13^ and inelastic^7, 14, 15^ neutron scattering. An overview of neutron scattering methods used to investigate phospholipid membranes was presented by Fragneto and Rheinstädter^16^, while Salditt focused on X-ray scattering^10^. *Neutron-Spin Echo* (NSE) measurements in quasi-reflective mode with a stack of supported membranes were introduced by Rheinstädter *et al*.^17^. Extending this approach, using only a single supported membrane stack, here we present GINSES as a novel method that enables the investigation of the dynamics in complex fluids in the vicinity of a macroscopic solid surface on a molecular scale. Comparing the two approaches, using only a single membrane stack, both the background from the surrounding D_2_O as well as the error in angle due to spatial separation of the single wafers is lower. This is especially true for extremely small *Q*_||_ (here ${Q}_{\parallel }=6.7\times {10}^{-3}$ Å^−1^), allowing the investigation of dynamics with a longer spatial range.

The basic principle is analogue to evanescent wave scattering of light^18, 19^, but on shorter length and time scales and with a different contrast, as given by the difference in interaction between matter and neutrons or photons. While GINSES as a method has been used before in our group^20–22^, only the use of the resonator enabled us to gain a clear enough signal in this special case and to properly distinguish for the first time between the in- and out-of-plane components of the scattering vector *Q*.

As an application, we show the direct measurement of the intra-membrane viscosity and the compressibility measurement of phospholipid membranes at the silicon/water interface. This was achieved by using resonance-enhanced GINSES to analyze the thermal fluctuations close to the solid interface on the scale of nanometers and nanoseconds. A multilayer structure (titanium-platinum) at the interface yields resonant enhancement of the evanescent neutron wave. This is essential to overcome the severe intensity limitation of the limited scattering volume ($\simeq 0.1\,\mu L$) filled by the evanescent field. These measurements grant access to properties of the membrane that have not been directly accessible until now. We demonstrate this measurement with a L-*α*-phosphatidylcholin (SoyPC) membrane that shows surface modes, which previously could not be observed by other methods. The structure of the above membrane close to the surface has been studied previously^20^ where the system was shown to develop a multilayer structure. Apart from indicating a promising novel method offering insight into the dynamics of complex fluids at solid/liquid interfaces, the results are also of immediate interest for the understanding of bio-lubrication in joints, where phospholipid membranes at interfaces contribute to the lubrication of joints^1^.

## Results

### GINSES

We performed a GINSES measurement on a stack of phospholipid layers at maximum swelling in D_2_O prepared on a silicon substrate. The structure of this system has been studied before using neutron grazing incidence small angle scattering (GISANS) and reflectometry^20^. The pure SoyPC in contact with liquid D_2_O at *T* = 35 °C forms double layers with a repeat distance of *d* = 68.4 Å. That repeat interval gives rise to the pseudo Bragg-peaks at $Q\simeq 0.092\,{\AA }^{-1}$.

In order to avoid influences on the dynamic measurement, such as de Gennes narrowing, the measurement was performed at $Q=0.11\,{\AA }^{-1}$ with an in-plane component of ${Q}_{\parallel }=Q\times \,\sin ([{\alpha }_{{\rm{in}}}-{\alpha }_{{\rm{out}}}]/2)=6.7\times {10}^{-3}$ Å^−1^ (see method section for details). Here the difference between incoming and outgoing angle of reflection was 7°.

**Figure 1:**
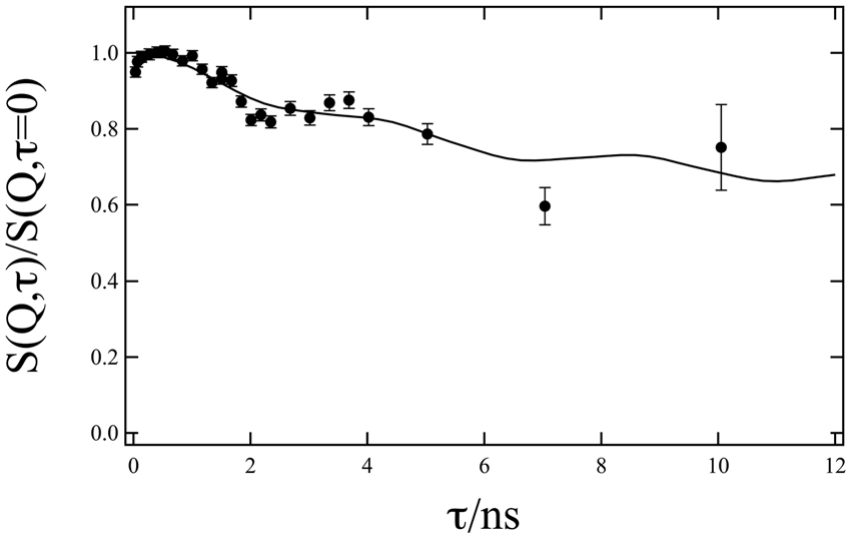
GINSES data of SoyPC membranes in aqueous solution at Q_||_ = 6.7 × 10^−3^Å^−1^. The solid line is a fit with the model from Romanov *et al*. as described in the text with *B* = 3.4 × 10^6^ Pa and ${\eta }_{3}=0.0075$ Pa · s.

This yielded the intermediate scattering function as shown in Fig. 1. We repeated the measurement with identical results (and better statistics) to confirm the results.

When compared to conventional NSE experiments on soft matter systems the most striking feature of the data presented in Fig. 1 is the apparent oscillation with a minimum at around 2 ns and a shallow (first) maximum at about 4 ns. For soft-matter samples on the typical NSE time- and length scales observation of underdamped motions is unexpected and very unusual due to the overwhelming dominance of friction compared to inertial forces on the low nm scale. These undulations only become visible on the longer in-plane length scale investigated here (${Q}_{\parallel }=6.7\times {10}^{-3}\,{\AA }^{-1}$ corresponding to ≈100 nm in real space).

We attribute these oscillations to undulation modes of the SoyPC membrane stack close to a solid substrate, which is corroborated by a model computation (solid line in Fig. 1) with values of $B=3.4\times {10}^{6}$ Pa for the compression modulus and ${\eta }_{3}=0.0075$ Pa · s for the in-plane viscosity of the membrane.

### Comparison with Simulations

**Figure 2:**
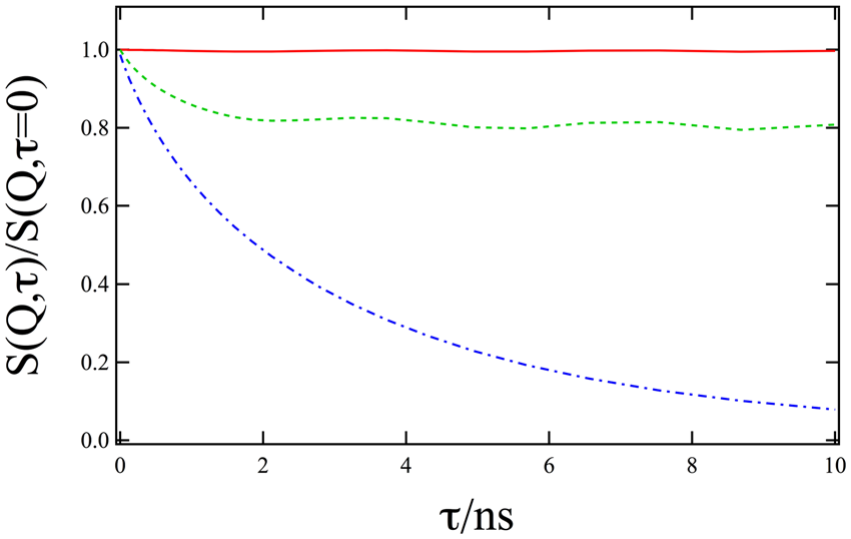
Calculation of the intermediate scattering function S(Q, t) with the Romanov model. The relaxation and oscillation of S(Q, t) is very sensitive to the in plane part, here with *Q*_||_ = 5.5 × 10^−3^ Å^−1^ (solid red line), 11 × 10^−3^ Å^−1^ (dashed green line), 16.5 × 10^−3^ Å^−1^ (dash-dotted blue line). All other parameters were kept constant at the values extracted from the fit to the data shown in Fig. 1.

In order to identify the nature of the undulations as observed in Fig. 1 we performed computations of the GINSES experiment based on the theory of Romanov *et al*.^23^. The corresponding results are shown in Fig. 2.

The model computations follow a membrane stack dynamics model by Romanov *et al*. applied to the present scattering situation of a soft-matter membrane adjacent to a solid substrate. Although the model in that publication was developed for use in liquid crystal multilayers, there is no restriction to its applicability to soft matter membranes using the correct parameters; indeed it has already been applied by Rheinstädter *et al*. to a similar system^16^.

Looking at the simulated data in Fig. 2 is obvious that such oscillations occur only for certain values of *Q*_||_ in the intermediate scattering function S(Q, t). We attribute this to the fact that for extremely small *Q*_||_ the membrane is seen on a larger scale, where only movements of the complete membrane would be visible. Similarly for large *Q*_||_ the local over-damped motion of a few phospholipid molecules is dominant. Thus, only in a certain range of *Q*_||_ are the undulations visible.

## Discussion

All modes observed here occur at solid-liquid interfaces, where a membrane is parallel and in close proximity to a solid substrate. Although very similar in nature, they must not be confused with capillary modes on free surfaces^24^ for freely supported films^25^, which have been observed in X-ray photocorrelation experiments.

A theoretical model to describe the dynamics of a finite lamellar system on a solid support in terms of layer undulation modes has been formulated by Romanov *et al*.^23^ applying the basic equation to describe smectic systems by Poniewierski^26^.

The model consists of *N* flexible layers in an equidistant stack on a solid support on the one end and a free surface at the other end of the stack. The layer-layer interaction is described in terms of the compression modulus *B* and the layer-bending elasticity in terms of a bending modulus *K* = *κ*/*d* (*κ*: Bending modulus, *d*: Thickness of the layers); both values are given as bulk properties and not for single layers. The model contains a surface tension *γ* for the free surface and the layer-layer sliding viscosity ${\eta }_{3}$. Eigenvalues for a mode *l* of the model solutions in ref. 23 represent layer-number *n* dependent displacements ${u}_{l}({\overrightarrow{r}}_{\parallel },n)$ with eigenfrequencies *ω*_*l*_. Each *ω*_*l*_ always contains a viscosity-dependent dampening term $-i{\eta }_{3}{q}_{\parallel }/(2\rho )$ and an additional term $\sqrt{f({q}_{\parallel },l)}$, with *f* also depending on the model parameters.

The modes are overdamped if *f* is negative. For a thick layer seen from below, where the evanescent wave is present, the surface tension *γ* only weakly influences the overall system, and intensity is contributed mainly by high *l* modes with sizeable displacement coefficients at the low-lying layers. With this (letting *l* = *N*) Eq. (2.19) used by Romanov *et al*.^23^ yields a condition for the occurence of oscillating modes:

1 $${q}_{\parallel } < {[\frac{16B\rho }{{d}^{2}{\eta }_{3}^{2}}]}^{1/4}$$

and the expression for the eigenfrequencies of the modes *l* is

2 $${\omega }_{\pm }^{(l)}=-i\frac{{\eta }_{3}{q}_{\parallel }^{2}}{2\rho }\pm \sqrt{\frac{4B}{\rho {d}^{2}}{\sin }^{2}\frac{(2l-1)\pi }{2(2N-1)}+\frac{4\gamma {q}_{\parallel }^{2}}{(2N-1)\rho d}{\cos }^{2}\frac{(2l-1)\pi }{2(2N-1)}+\frac{K{q}_{\parallel }^{4}}{\rho }-\frac{{\eta }_{3}{q}_{\parallel }^{4}}{4{\rho }^{2}}}.$$

Here $K=\frac{\kappa }{d}$. Inserting typical values ($B={10}^{6}\,{\rm{J}}/{{\rm{m}}}^{3}$, $\rho ={10}^{3}\,{\rm{k}}g/{{\rm{m}}}^{3}$, ${\eta }_{3}={10}^{-2}\,{\rm{kg}}/({\rm{ms}})$, $d=6\times {10}^{-9}\,{\rm{m}}$) yields ${q}_{\parallel } < \,1.5\times {10}^{-7}\,{{\rm{m}}}^{-1}=1.1\times {10}^{-3}\,{\AA }^{-1}$, well below the typical *Q* values of NSE but in the range of *Q*_||_ of GINSES experiments. Note, however, that *q*_||_ denotes a *mode wavevector*, which is different from the scattering wavevector *Q*_||_. Certainly, matching mode wavevectors make a significant contribution to the scattering intensity observed at a corresponding value of *Q*_||_ but there is no strict one-to-one correspondence and the coupling is such that other modes do contribute. This is also why the expression for the eigenfrequency cannot be employed directly, as only an integration over all possible eigenmodes will render a usable result for any given *Q*_||_ value.

The GINSES geometry has another feature that favours the observation of undulation modes in the present case of lamellar stacked layers. The layer displacements $\overrightarrow{u}$ along the z-direction (perpendicular to the interface) are in the direction of the main component of the scattering vector $\overrightarrow{Q}$. Since the signal intensity basically depends on the projection of $\overrightarrow{Q}$ and $\overrightarrow{u}$ on each other ($I \sim {(\overrightarrow{Q}\cdot \overrightarrow{u})}^{2}$) it benefits from the much larger perpendicular $\overrightarrow{Q}$-component.

**Figure 3:**
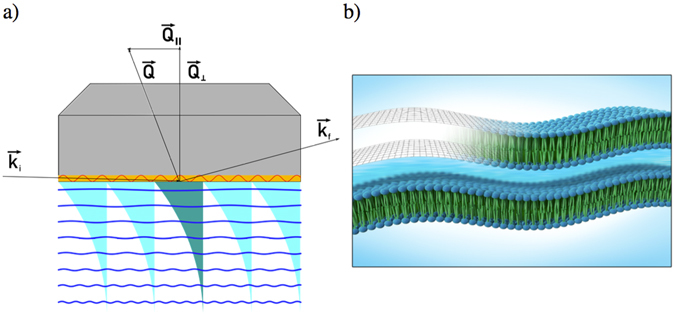
(**a**) Depiction of the geometry of a GINSES experiment. Unlike reflectometry, the incident and outgoing angle differ, which means the total Q-vector is not perpendicular to the surface. Note: the incoming neutron beam passes through the Si-block before scattering on the phospholipid membrane. The intensity of the evanescent wave penetrating the sample is shown as a blue exponentially decaying wave into the sample. In order to illustrate the effect of the resonator (yellow layer) the standing neutron wave (red sinusoidal line) is shown. The evanescent wave in the absence of the resonator is shown in a darker colour, the additional intensity is shown by the lighter evanescent waves penetrating the sample layers (blue wavy lines). (**b**) Sketch of the sample structure.

The compression modulus *B* and the in-plane viscosity ${\eta }_{3}$ have proven to be the governing parameters of the relaxation behaviour of membranes close to a solid substrate (see Fig. 3, Methods). While the compression modulus *B* is also accessible by other methods, such as X-ray reflectometry^6, 10, 27, 28^ it has not been previously possible to directly measure the in-plane viscosity within this time and size regime (nanoseconds and nanometers) as well as possible deviations of the interface layer compression modulus from the bulk value in the vicinity of a solid substrate. Here it should also be mentioned, that using the Caillé exponent approach^29^ as used in some of the other publications is an indirect method, as compared to the method presented here. This is due to the fact that the Caillé theory is based on elastic energies, independent of the viscosity, whereas the modes presented here depend on both.

Comparing the simulations in Fig. 2 and the actual measurement data in Fig. 1 there are two main observations to be made: (1) There are indeed undulations visible, with a similar behaviour in terms of frequency and amplitude as measured by GINSES and (2) these undulations vanish at high in-plane Q_||_, indicating that the surface modes detected here are indeed long range correlations.

Here, long-range in-plane modes of phospholipid membranes adjacent to a solid substrate-liquid interface are observed. While not completely dissimilar they should be clearly distinguished from surface modes at liquid-gas interfaces^24^ as well as internal modes of membranes^17^.

In Table 1 a comparison is given between literature values and values obtained from the fit shown in Fig. 1. These parameters were determined using quasi-elastic neutron scattering^16^, using a Langmuir trough with a tensiometer^30^ and driving a lipid vesicle through an aperture^31^, so they give a representative sample of both available techniques and possible values for the parameters.

**Table 1:** Comparison between literature values and values obtained from a parameter fit as shown in Fig. 1. *γ* was adjusted manually to obtain a stable fit.

	Literature value	Fitted value
*B*/Pa	1.1 · 10^7^ ^16^	3.4 ± 0.4 · 10^6^
*γ*/*N* · *m*	0.045^30^	0.01
${\eta }_{3}/Pa\cdot s$	0.016^16^	0.0076 ± 0.0009
$\kappa /{k}_{B}\cdot T$	19.5^31^	22.4 ± 1.2

A more detailed comparison shows that the difference between the various modes of measurement is quite pronounced. While the bending properties signified by $K=\kappa /d$ as a collective motion of an assembly of phospholipid molecules is virtually identical, the intra-membrane viscosity ${\eta }_{3}$ is reduced. This reduction can be explained by looking at the length scales involved in these measurements. While the experiment performed by Rheinstädter *et al*. is performed for ${Q}_{\parallel }\le 1\times {10}^{-2}$ Å^−1^ the experiment shown here is performed for ${Q}_{\parallel }=6.7\times {10}^{-3}$ Å^−1^. This translates to length scales of ≤500 Å for Rheinstädter *et al*. and ≥1000 Å for the experiment shown here. This allows the conclusion that the local viscosity is higher than the one averaged over a larger area of the membrane.

The increase in the compression modulus *B* can also be explained by similar considerations: a membrane may be much more rigid locally, than the same membrane averaged over a large area, because the necessary displacement for each single phospholipid molecule becomes increasingly smaller as the number of molecules participating the in motion increases. Additionally the vicinity of the hard substrate may inhibit fluctuations that would decrease the compression modulus *B*.

Surface tension was adapted manually to our fit. Here it should be stated that surface tension at the air-water interface as investigated by Brzozowska *et al*.^30^, may fundamentally differ from the surface tension of a layer stack and that thus a variation is to be expected. Rheinstädter *et al*.^16^ argued that for such a case the surface tension might even be neglected completely. In addition, *γ* is only considered for the topmost layer of the membrane stack^23^.

In terms of a theoretical prediction of the observed surface modes, Gompper *et al*.^32^ also predicted a damped oscillating correlation function for a bulk microemulsion model on a different basis $S(Q,\tau )/S(Q,\tau =0)=$ $(A-1)\exp {({{\rm{\Gamma }}}_{1})}^{\beta }+A\exp ({{\rm{\Gamma }}}_{2})\cos (\omega \tau )$, however with no direct link to the parameters *B*, *γ*, ${\eta }_{3}$ and *κ* and the relevant length scale. Our present result indicates that this predicted behaviour indeed occurs, but only at the *Q*-values probed here, conforming with the results of the simulation.

The the analysis of these modes and the resulting knowledge of the rheological key parameters of phospholipid membranes may prove useful in applications in biotechnology and medical sciences. One very prominent example is in phospholipid membranes on a solid substrate in an aqueous environment as found in mammalian joints^1^.

Any considerations given here about the interplay between the described mechanism and the stability of a given membrane are very general. The investigated membrane is structurally very similar to the membranes found in mammalian joints^1^. We did not encounter this behaviour in any softer systems we investigated previously, such as those in microemulsions^22^. From general observations it is clear that the membranes withing mammalian joints need to be durable (individuals can reach 100 years of age), compared to those in emulsions, which exist for a time period of mere seconds or minutes. Thus a self-stabilizing mechanism must exist in these membranes, and such an undulation as reported here is an excellent candidate for dissipating energy throughout the system. The stability of a membrane is also linked to the stability as described by Salditt^10^; however in this case phase transitions due to temperature change probably play a major role.

## Methods

### Experimental setup

Neutron scattering experiments were performed using neutron spin-echo spectrometer at the spallation neutron source SNS at the Oak Ridge National Laboratory^33^. Neutron spin echo (NSE) spectroscopy provides the highest energy resolution in neutron scattering and measures the normalized time correlation function in reciprocal space, S(q, *τ*). NSE uses a polarized neutron beam.

The high resolution is achieved by encoding and decoding the neutron velocity before and after the scattering process by way of a number of spin precessions in a magnetic field. In grazing incidence geometry, the sample cell is very similar to that used in neutron reflectometry experiments. The incoming neutron beam hits the interface between a Si-block and the sample containing the phospholipid membranes at an angle below the critical angle, thus being totally reflected. Only an evanescent wave penetrates the sample and can be scattered by the membranes in the vicinity of the interface. Only the near-interface dynamics are probed in this geometry. The penetration depth of the evanescent wave can be varied by changing the incident angle and the contrast between sample and Si-block (by an appropriate deuteration of the sample). In order to overcome the huge intensity penalty due to the strongly collimated beam and the use of the evanescent wave only, a resonator structure on top of the Si-block, consisting of Pt-Ti layers at the interface, which provide a neutron waveguide for an appropriate incident angle, has been used. The intensity of the evanescent wave very close to the interface is increased in this improved GINSES setup.

The sample stage consists of a polished block of single crystalline silicon with a Ti-Pt multilayer between the silicon and the sample (see Fig. 3). The Ti-Pt layer structure forms a resonator that enhances the evanescent wave intensity between one and two orders of magnitude in a narrow range of selected values of *α*_i_ values. This is analogue to the use of dielectric layers in light optics^34^. The fluid sample is contained in a volume confined by this interface and a glass plate at a few 0.1 mm distance, sealed by O-rings^20^. The active area was 2 × 15 cm^2^, while the Si-block was 5 cm thick.

The sample cell was used in a reflectometer-type geometry. Unlike in typical NSE experiments, the beam was narrowly collimated in the direction of the scattering plane down to a divergence of ±0.05° by a slit at the entry of the neutron beam to the secondary spectrometer and a slit attached to the front side of the Si-plate. The orientation of the Si-block was such that the collimated beam hit the interface at an angle below the critical total reflection angle ${\alpha }_{c}=\lambda \sqrt{{\rm{\Delta }}\rho /\pi }$ (where Δ*ρ* is the scattering length density difference between the sample and Si-block) so that there is only the narrow evanescent neutron wave on the sample side. In this case an incoming angle of *α*_*i*_ = 0.02° was chosen. The direct beam is absorbed by a cadmium strip on the exit side of the Si-block. Evanescent field neutrons that are scattered by the sample reenter Si-block and are transmitted with an average angle of several degrees. They undergo the second part of the NSE-typical Larmor precession velocity encoding-decoding analysis and are detected from within a solid angle of ΔΩ = 4° × 4°. The intensities are evaluated to yield the intermediate scattering function S(*Q*, *t*) on detector slices with a solid angle of ΔΩ = 1° × 4°.

Since the SNS-NSE instrument is located at a pulsed spallation source it uses a broad range ${\rm{\Delta }}\lambda \simeq =3\,\AA$, both critical angle and penetration depth of the evanescent wave would usually be ill-defined. In order to compensate for this effect a MgF_2_ prism with an apex angle of 170° was used. This establishes an angle-wavelength correlation such that the penetration depth of the evanescent wave ${\rm{\Lambda }}=\mathrm{1/}\sqrt{4\pi {\rm{\Delta }}\rho \mathrm{(1}-{\alpha }_{{\rm{in}}}^{2}/{\alpha }_{{\rm{c}}}^{2})}$ approximately stays constant in the range of ${\rm{\Lambda }}\simeq 500\,\AA$ ^22^. We observe a finite number (on the order of 10) of double layers due to the finite penetration depth.

A sketch of the used geometry is shown in Fig. 3. While the incident beam is at a fixed angle with respect to the substrate (0.2° in front of the prism in our case), the position of the detector and thus the exiting angle varies. The primary incident wave-vector ${\overrightarrow{k}}_{{\rm{i}}}$ undergoes total internal reflection at the interface to the resulting final beam ${\overrightarrow{k}}_{f}$.

The classical reflectometry condition of ${\alpha }_{i}={\alpha }_{f}$ with the first being the incoming and the latter being the outgoing angle of reflection is not satisfied here. This gives rise to an in-plane component of $\overrightarrow{Q}={\overrightarrow{k}}_{i}-{\overrightarrow{k}}_{f}$, namely ${Q}_{\parallel }=Q\times {\rm{s}}{\rm{i}}{\rm{n}}([{\alpha }_{{\rm{in}}}-{\alpha }_{{\rm{out}}}]\mathrm{/2})$ as shown in Fig. 3.

As the difference between the incoming and outgoing angle of just a few degrees is still small, this implies very small in-plane components of $\overrightarrow{Q}$, on the order of 10^−3^ Å^−1^. In the present experiment the deviation between the incoming and outgoing beam was 7°. This allows to calculate of the in-plane component of the Q-vector with the expression ${Q}_{\parallel }=Q\times {\rm{s}}{\rm{i}}{\rm{n}}([{\alpha }_{{\rm{in}}}-{\alpha }_{{\rm{out}}}]\mathrm{/2})$. With a total Q-value of $Q=0.11\,{\AA }^{-1}$ this yields an in-plane component of ${Q}_{\parallel }=6.7\times {10}^{-3}\,{\AA }^{-1}$. To our knowledge, this is the first report of such small Q-values being achieved in any NSE experiment. Also - except for the angle of total reflection - we ignored the difference in neutron refraction indices between sample, Si and vacuum/air, which is justified since the outgoing scattered radiation hits the interfaces at several degrees.

### Resonator

In previous experiments we already performed GINSES measurements; however, the extremely low sample volume limited the experiments to strongly scattering samples^20, 21^. Even under optimal conditions it was not always possible to achieve the necessary count rates and, by extension, the accuracy of the intermediate scattering function, for the analysis presented here. The intensity limitations can be alleviated by using a resonator structure on top of the Si-block, which acts as a neutron wave-guide and increases the field of the evanescent wave. Three double layers of titanium (130 Å) and platinum (320 Å) and a final layer of titanium (130 Å) provide a SLD profile that leads to three conditions of constructive interference for angles below the angle of total critical reflection. In Fig. 3 this is indicated by one dark evanescent wave that is achieved in the setup without the resonator, and the additional, lighter evanescent waves that can be excited in the presence of a resonator.

Constructive interferences were obtained while evanescent waves developed in the sample, so that the intensities were accumulated. For sharp conditions of resonance, amplification factors of 10 to several 100 can be obtained. Under conventional collimation conditions, the gain factors are still between 3 and 10. This gain was essential for the observations of the collective membrane modes observed here due to the small amplitudes of the individual phospholipid bilayer.

## Material

For the preparation of the sample we followed the protocol from our previous publication^20^. 15 *mL* of SoyPC/isopropanol solution with a concentration of 174.6 *mgmL* ^−1^ were prepared. The solution was shaken for at least 15 min. until a clear solution was obtained. This solution was subsequently deposited on top of a silicon block in a holding frame, which had been previously cleaned with a 2% Hellmanex solution and rinsed with Milipor water. Afterwards the sample was dried overnight at room temperature and a pressure of 2500 Pa in order to prevent bubbles. This preparation method yielded macroscopically thick films on the Si-block. To facilitate measurements, the sample cell was kept at 35 °C and was completely filled with D_2_O in order to obtain a completely hydrated multilayer sample.

Although the penetration depth of the neutrons into the sample is only a few hundred nanometers, we used a preparation protocol that renders a film thickness in the millimetre range. Earlier experiments with thinner films (approximately 20% of the material on the same surface area) have shown that this does not change the behaviour of the film close to the substrate, but it facilitates sample preparation and reproducibility.

### Data analysis

The standard neutron spin echo data evaluation consists in precisely determining the degree of polarization of the neutron beam at the detector and normalizing it with the degree of polarization of a reference measurement with an elastically scattering sample (graphene foil).

The intermediate scattering function determined in this way, S(q, *τ*), has been fitted with a model for the scattering function described by Romanov *et al*.^23^ extended to include the dynamics of the system as described previously.

#### Background considerations

Incoherent background scattering in this experiment can be accounted for in two ways: hydrogen in the phospholipid itself as well as the incoherent contribution from the solvent D_2_O.

Since the interface region also contains a significant amount of hydrogen (within the phospholipid layer), is confined in the membrane, it will reduce the polarization of the echo measurement as well as for the elastic polarization measurement. Long wavelength undulations cannot be observed with the incoherent part of the scattering, since it represents only the self-correlation function, not the pair-correlation function. This part has been included as a static incoherent contribution in the model, which thus only accounts for the hydrogen in the phospholipid itself.

Secondly, the solvent (D_2_O) also contributes to the incoherent scattering (*p*(spin incoherent scattering) = $\frac{1}{3}$). This is mainly visible as the initial increase of the scattering function for Fourier times *τ* < 0.5 ns. The relaxation time ${\tau }_{0}=1/(D{Q}^{2})$ is fitted with a diffusion constant *D* = 190 Å^2^/ns which we would expect for pure deuterated water diffusion. The incoherent contribution from the solvent D_2_O seems to be slightly higher than expected compared to the phospholipid layer contribution, which might stem from some residual protonated solvent molecules from the sample preparation process.
